# Book Notices

**Published:** 1858-01

**Authors:** 


### BOOK NOTICES.

LECTURES ON THE DISEASES OF WOMEN. By CHAS. WEST, M.D., Fellow of the Royal College of Physicians, Examiner in Midwifery at the Royal College of Surgeons, England, Physician-Accoucheur to St. Bartholomew Hospital, and Physician to the Hospital for Sick Children. Part 1st—Diseases of the Uterus. Philadelphia: Blanchard & Lea, 1857. For sale by Keen & Lee, Chicago.

"In estimating the value of the speculum as a means of diagnosis, I think that the advances in our knowledge of uterine diseases of which it was the indirect occasion, by the impulse it gave to their study, we sometimes confound with those positive additions to our information which we owe exclusively to the use of that instrument. The former have been very great indeed, and I think candor compels us to acknowledge that they have been due almost exclusively to persons who, not content with our previous means of investigating uterine disease, have labored to increase them by the employment of instruments. The latter have certainly been less considerable, but nevertheless the speculum enable us in many instances to decide at once and with certainty, upon the nature of a case, which otherwise we should have understood only after long and careful watching, to discover some minute polypus which the fingers alone would not have detected, to determine the source of a profuse leucorrheal discharge, and to decide whether it is furnished by the cavity of the womb or the walls of the vagina, or from the redness, congestion, or abrasion of the os uteri, to infer the state of the womb generally, and thus to conduct our treatment upon the sure ground of positive observation, not upon bare presumption. At the same time, however, that I hold the speculum to be in many cases of most essential service, I think that the endeavor of all of us should be to ascertain the minimum of frequency with which its employment is necessary. That is to be done not by decrying the instrument, still less by attributing dishonest motives to those who use it, but by soberly and honestly trying to test the value of the information we derive from it, and learning to discriminate between those appearances which the speculum discloses that are of moment, and such as are of no importance." (Page 33.)

The above is Dr. West's estimate of the speculum. His position with regard to the use of it and local applications to the uterine neck may be considered conservative, and all his opinions and treatment as detailed in this little volume are characterized by acute discrimination and temperate energy. Its contents are divided into twenty lectures,—beginning with the symptoms, mode of examination and importance that should be attached to disease of the uterus, passing through the lighter up to the more grave functional disorders of the female sexual system, displacements of the uterus, non-malignant organic diseases of structure, and ends with a graphic description of cancerous affections. Nothing short of a patient and thorough examination of the work itself, will enable the reader to form a just estimate of the amount of valuable and available information it contains. There are no flourishes, or an egotistical display, of originality to the prejudice of his predecessors or compeers, but a plain, straight-forward and philosophical exhibition of the state of our knowledge on the subjects of which he treats. We know of no treatise of the kind so complete and yet so compact.

---

PNEUMONIA, its Pathology and Treatment. By EZRA READ, M.D., of Terre Haute, Ind. From the *Nashville Journal of Medicine and Surgery*. Nashville: Cameron & Fall.

To all who are acquainted with our accomplished friend, Dr. Read, it would be needless to say that this essay is replete with sound sense, and information. Dr. Read describes a species of pneumonia with which we are personally well acquainted, curable by quinia alone. Such publications are necessary occasionally to open our eyes to some things that are transpiring around us unobserved.

---

GENERAL THERAPEUTICS AND MATERIA MEDICA, adapted for a Medical Text Book with Indexes of Remedies and of Diseases and their Remedies. By ROBLEY DUNGLISON, M.D., L.L.D., Professor of Institutes of Medicine, etc., in the Jefferson Medical College of Philadelphia, formerly Professor of Materia Medica and Therapeutics in the University of Virginia and Maryland and in Jefferson Medical College of Philadelphia; with one hundred and ninety-three illustrations. Sixth edition, revised and improved, in two volumes. Philadelphia: Blanchard & Lea, 1857. Pages 544 and 539.

The profession is too well acquainted with this work to require any comment from us. It is sufficient to say that this edition is "improved," as the author assures us. For sale at the book store of Keen & Lee, Chicago.

---

A THEORETICAL AND PRACTICAL TREATISE ON MIDWIFERY: Including the Diseases of Pregnancy and Parturition and the attentions required by the child from birth to the period of weaning. By P. CAZEAUX, Member of the Imperial Academy of Medicine, Adjunct Professor in the Faculty of Medicine of Paris, Chevalier of the Supplementary Number of the Order of Charles III., Member of the Surgical Society, of the Biological Society, of the Medical Society of Emulation, of the Anatomical Society, Non-Resident Associate of the Medical Society of Bordeaux, Correspondent of the Society of Accoucheurs of Berlin, President of the Medical Society of the department of the Seine. Adopted by the Superior Council of Public Instruction, and placed by ministerial decision in the rank of the classical works designed for the use of Midwifery Students in the Maternity Hospital of Paris. Second American from the fifth French Edition. Translated by Wm. R. Bullock, M.D., with one hundred and forty illustrations. Philadelphia: Lindsay & Blakiston, 1857. For sale by Keen & Lee, Chicago.

We should regret the necessity (want of space) which compels our notice of this truly classical work into brief dimensions, if we did not know that the profession throughout this, and in fact all civilized communities, are well acquainted with its merits. This edition is a noble specimen of authorship profusely complete in every department of obstetrics, without useless prolixity. Too much praise cannot be awarded the talented translator for the faithfulness of his task, and the easy flow he has given, to the rendering of the text from the French to our own language. Its typography is brilliant and very correct, as is the case with the books of Lindsay & Blakiston generally. It would but be doing justice to the book, and but the expression of our honest conviction, after a careful examination, to say that we know of no work on this all important branch of our profession that we can recommend to the student or practitioner as a safe guide through the perils and perplexities of the lying-in month before this.

---

Introductory Lecture, delivered by D. Warren Brickell, M.D., Professor of Obstetrics, N. O. School of Medicine. Nov. 3, 1857. Published by request of the Class. New Orleans, La.

Address: Introductory to the Fifteenth Annual Course of Lectures in Rush Medical College. Delivered Nov. 2d, 1857. By N. S. Davis, M.D., Professor of Principles and Practice of Medicine in Rush Medical College, Chicago, Ill.

The above addresses, in style and character of contents, are fair representatives of the almost antipodal differences of their two talented authors. B.

---

Medical Lexicon: A Dictionary of Medical Science, containing a concise explanation of the various subjects and terms of Anatomy, Physiology, Pathology, Hygiene, Therapeutics, Pharmacology, Pharmacy, Surgery, Obstetrics, Medical Jurisprudence, Dentistry, etc.; Notices of Climates and of Mineral Waters; Formula for Officinal, Empirical, and Dietetic Preparations, etc., with French and other synonyms. By Robley Dunglison, M.D., L.L.D., Professor of the Institutes of Medicine, etc., in the Jefferson Medical College of Philadelphia. Revised and greatly enlarged. Philadelphia: Blanchard & Lea, 1857. Pages 992. For sale by Keen & Lee, Chicago.

Six thousand subjects and terms have been added to the contents of the last edition. This is the fifteenth edition and contains in all 60,000 subjects and terms. We know of no book with which to compare this but Webster's unabridged dictionary. It is as indispensable to the intelligent physician as Webster is to the general scholar. Both now are the standard dictionaries of the English language, and received universally as authorities throughout the civilized world. In this respect, at least, young America can boast of successful competition with the world; she has produced the two best dictionaries of the age.

---

We have received a copy of the TRANSACTIONS OF THE AMERICAN MEDICAL ASSOCIATION for 1857, containing the Minutes, the Report of the Treasurer, Casper Wistar, the Address of President, Zina Patcher; Report on the Medical Topography and Epidemics of Maryland, by E. G. Waters, M.D.; Report on Infant Mortality in large cities, the sources of its increase and means for its diminution, by D. Meredith Reese, M. D., L.L.D., etc., of New York; Report on the Medico-legal Duties of Coroners, by Alexander J. Semmes, M.D.; Report on the Topography and Epidemic Diseases of the State of Georgia, by John F. Posey, M.D., of Savannah; Report on the Use of Cinchonia in Malarious Diseases, by E. Hinkle, M.D.; Report on the Blending and Conversion of types in Fevers, by C. G. Pease, M.D., Janesville, Wisconsin; Report on a New Principle of Diagnosis in Dislocations of the Shoulder Joint, by L. A. Dugas, M.D., Professor of Surgery in the Medical College of Georgia; Report on the Facuna and Medical Topography of Washington Territory, by G. Suckley, M.D., U.S. Army, Fort Steilacoon, W. T., Oct. 1, 1856; Report on the Medical Flora of Washington Territory, by E. G. Cooper, M.D., 1857; Report on Deformities after Fractures, by F. Hastings Hamilton, M.D., Part Third, (this is a lengthy and very important report); Partial Report on the Nervous System in Febrile Diseases, by Henry F. Campbell, M. D., of Georgia; Prize Essay; The Excito-Secretory System of Nerves, its relation to Physiology and Pathology, by Henry Fraser Campbell, M.D., Professor of Special and Surgical Anatomy in the Medical College of Georgia, etc. etc.; "Observation becomes Experiment, when used in severe processes of induction," Victor

Cousin, May, 1857; Prize Essay; Experimental Researches relative to the Nutritive Value and Physiological Effects of Albumen, Starch and Gum, when singly and exclusively used as Food, by Wm. A. Hammond, M.D., Surgeon U. S. A.; Plan of Organization of the American Medical Association; Code of Ethics of the American Medical Association, adopted May, 1857; Catalogue of Officers and Permanent Members of the American Medical Association.

The mechanical execution of this volume is done in Collins' best style, and makes a very respectable appearance. As will be seen, by examining the volume, the papers are nearly all short and very practical. The report of Prof. Dugas, which is all contained in less than five pages, is worth more than the cost of the whole volume of the transactions to any practising physician. His principle of diagnosis is so striking, and expressed in such short style, that we cannot refrain from copying it. "If the fingers of the injured limb can be placed by the patient or the surgeon upon the sound shoulder, while the elbow touches the thorax, there can be no dislocation. In other words, it is physically impossible to bring the elbow in contact with the sternum, or point of the thorax, if there be a dislocation, and the inability to do this is proof positive of the existence of dislocation; inasmuch, as no other injury of the shoulder-joint can induce this inability." Any physician can demonstrate the correctness of this principle on the skeleton to his entire satisfaction, in a very few minutes. By sending three dollars and his address to Casper Wistar, M. D., Philadelphia, any person can procure a copy of the Transactions by mail.

---

TRANSACTIONS OF THE ILLINOIS STATE MEDICAL SOCIETY FOR THE YEAR 1857. Chicago: Chas. Scott & Co., Book and Job Printers.

These Transactions have been thus late in making their appearance, from the fact, as Dr. Johnson, the Permanent Secretary, informs us, that the different papers were not furnished by their respective authors to the publishing committee until within a few weeks past. The next meeting of the Society will be held at the city of Rockford, on Tuesday, June 1st, 1858.

The papers contained in the "Transactions" are: The Address of the President, H. Noble, M.D., of Independence; Report of the Committee of Practical Medicine, by C. N. Andrews, M.D., of Rockford; Report of the Committee on Drugs and Medicines, by H. A. Johnson, M. D., of Chicago, Chairman; Report of Committee on the Properties of the Asclepias Tuberoso, by C. Goodbrake, M.D., of Clinton; Report on Congestive Intermittents, by Dr. F. K. Bailey, of Joliet; Report on the Changes which take place in the Blood in the Continued Forms of Fever, by N. S. Davis, M.D., of Chicago; Stomatis Materna, read before the Esculapian Society, at the meeting in Charleston, Illinois, May 27th and 28th, 1857, and ordered to be communicated to the State Medical Society, by W. M. Chambers, M.D., of Charleston; Cases of Severe Mechanical Lesions of the Knee Joint Successfully Treated, by Calvin Truesdale, M.D., Rock Island. At the end of the volume, the names of all the members of the Society are arranged in alphabetical form.

It would afford us much pleasure to enter into a critical review of these contributions, more especially as we find much in them that is valuable and worthy of extensive dissemination, and they are the productions of men exercising their profession in our own State, but space will not permit it. At some future time we expect to make such extracts as will set forth the prominent features of the more practical of these papers. The authors have all shown capacity and industry, of which the profession of the State have just reason to be proud.

---

Journal de Physiologie de l'Homme et des Animeaux. Par le Docteur E. Brown-Sequard. (Journal of Physiology of Man and Animals. By E. Brown-Sequard, M. D.)

A Quarterly Journal, with the above title, is to be issued in Paris, commencing January 1, 1858. Each number will contain from 160 to 200 pages, with plates and figures interspersed in the text. There can be no doubt of the great utility of a journal devoted to physiology and the kindred sciences. Their rapid progress and bearing upon practical medicine and surgery, as well as medical jurisprudence, render them at the present moment objects of public interest. It is indeed not a little singular that while in the German language, besides the celebrated *archives* of Muller, there are five periodicals devoted in a great measure to physiology, there is not at the present time one in either the French or English languages in which this is now made a leading object.

It is scarcely necessary to say that Dr. E. Brown-Sequard is the man for such a work. Familiar with not only the works of all the most eminent writers in French, English and German, but acquainted personally with the authors themselves; learned beyond any living physiologist in France, England or America; original beyond any except Cl. Bernard, whom he fairly rivals; enthusiastic and devoted to the last degree to his pursuits; we are justly entitled to expect that the appearance of his Physiological Journal will constitute a new era in physiological science.

The price of the Journal in this country will be *five dollars*, payable in advance. Dr. Brainard, of this city, will take charge of remittances and transmit orders if desired. Barrington of Philadelphia is the American agent.

